# A radiosensitizing effect of RAD51 inhibition in glioblastoma stem-like cells

**DOI:** 10.1186/s12885-016-2647-9

**Published:** 2016-08-05

**Authors:** Anaïs Balbous, Ulrich Cortes, Karline Guilloteau, Pierre Rivet, Baptiste Pinel, Mathilde Duchesne, Julie Godet, Odile Boissonnade, Michel Wager, René Jean Bensadoun, Jean-Claude Chomel, Lucie Karayan-Tapon

**Affiliations:** 1 INSERM1084, Laboratoire de Neurosciences Expérimentales et Cliniques, Poitiers, F-86021 France; 2 Université de Poitiers, U1084, Poitiers, F-86022 France; 3 CHU de Poitiers, Laboratoire de Cancérologie Biologique, Poitiers, F-86021 France; 4 CHU de Poitiers, Service d’Oncologie Radiotherapique, Poitiers, F86021 France; 5 CHU de Poitiers, Service d’Anatomo-cytopathologie, Poitiers, F86021 France; 6 CHU de Poitiers, Service de Neurochirurgie, Poitiers, F86021 France

**Keywords:** Glioblastoma stem cells, RAD51, Radioresistance, Comet assay

## Abstract

**Background:**

Radioresistant glioblastoma stem cells (GSCs) contribute to tumor recurrence and identification of the molecular targets involved in radioresistance mechanisms is likely to enhance therapeutic efficacy. This study analyzed the DNA damage response following ionizing radiation (IR) in 10 GSC lines derived from patients.

**Methods:**

DNA damage was quantified by Comet assay and DNA repair effectors were assessed by Low Density Array. The effect of RAD51 inhibitor, RI-1, was evaluated by comet and annexin V assays.

**Results:**

While all GSC lines displayed efficient DNA repair machinery following ionizing radiation, our results demonstrated heterogeneous responses within two distinct groups showing different intrinsic radioresistance, up to 4Gy for group 1 and up to 8Gy for group 2. Radioresistant cell group 2 (comprising 5 out of 10 GSCs) showed significantly higher RAD51 expression after IR. In these cells, inhibition of RAD51 prevented DNA repair up to 180 min after IR and induced apoptosis. In addition, RAD51 protein expression in glioblastoma seems to be associated with poor progression-free survival.

**Conclusion:**

These results underscore the importance of RAD51 in radioresistance of GSCs. RAD51 inhibition could be a therapeutic strategy helping to treat a significant number of glioblastoma, in combination with radiotherapy.

**Electronic supplementary material:**

The online version of this article (doi:10.1186/s12885-016-2647-9) contains supplementary material, which is available to authorized users.

## Background

Radiotherapy is a treatment modality for glioblastoma (GBM) in combination with surgery and chemotherapy (Stupp protocol). However, GBM are resistant to current treatment with recurrence patterns and a median survival of 14.6 months [1]. It is now well-established that GBM are composed of heterogeneous tumor cell populations, including tumor cells with characteristics similar to neural progenitor cells called “glioblastoma stem cells” (GSCs) [2, 3]. Accumulating evidence indicate that GSCs can survive DNA damage and are able to repopulate the tumor after treatment [4, 5] contributing to radioresistance and tumor recurrence. Initial reports have linked the stemness properties of GSCs to CD133 expression and suggested that tumorigenic cells in GBM were restricted to the CD133^+^ population [3]. Bao et al. reported that compared to CD133^−^ cells, CD133^+^ cell exposure to ionizing radiation (IR) increased colony-formation efficiency and decreased apoptosis level. The better survival of CD133^+^ cells was attributed to preferential activation of the G2/M DNA-damage checkpoint response and increased DNA repair capacity compared with normal cells [4]. Studies from McCord et al. corroborate these results but clearly indicate that the expression of CD133 is not associated with the radioresistant phenotype of GSCs when compared with unsorted glioma cell lines [6]. More recently, Fouse et al. reported a lack of association between the extent of CD133 expression and response to radiotherapy in a patient-matched study [7].

Several radioresistance mechanisms have been identified in GSCs, such as better efficiency of DNA repair systems [4, 8, 9], preferential activation of the G2/M DNA-damage checkpoint response [4], a higher level of anti-apoptotic factors [5], and sustained expression of pluripotency factors such as Notch [10] or Signal Transducer and Activator of Transcription 3 (STAT3) [11]. Recently, Dahan et al. reported that IR were able to induce the dedifferentiation of glioblastoma cells to a stem-like phenotype that may contribute to radioresistance [12]. Studies from Jamal et al. have demonstrated that tumoral and brain microenvironments influence GSCs radioresponse, and notably GSCs under intracerebral growth conditions were more radioresistant than in vitro [13, 14]. DNA double-strand breaks (DSB) are the main cytotoxic lesions induced by ionizing radiations (IR). In the absence of efficient DSB repair mechanisms, extensive DNA damage can lead to cell death. DSB response is a multi-step process consisting in damage sensing, signal transduction to the repair complexes, cell cycle arrest, and induction of apoptosis. Two major pathways are involved in DSB repair: non-homologous end joining (NHEJ) and homologous recombination (HR) [15]. Previous reports have indicated that IR-exposed fibroblasts preferentially activate the HR pathway [16]. In a similar manner, unlike neural progenitor cells using the NHEJ pathway, GSCs preferentially activate the HR pathway to repair DNA damage [8, 17, 18].

It is now generally considered that GSCs contribute to GBM radioresistance, and are a critical target in efforts to improve therapeutic outcome. Therefore, complete eradication of GSCs is necessary to obtain sustained disease remission. In this respect, an effective treatment approach would selectively sensitize GSCs to IR, requiring the identification of new therapeutic targets. For this purpose, ten GSC lines derived from patients with primary GBM have been isolated and established in culture. These cells had a capacity for proliferation, self-renewal and differentiation, recapitulating the phenotype of the tumor from which they were derived. In addition, all GSC lines were able to generate tumors in immunodeficient mice [11, 19, 20]. In our previous reports we characterized their stemness properties and analyzed gene expression features associated with their tumor-initiating properties [11, 19–21]. In this study, we have analyzed the DNA damage response after IR in 10 primary GSCs lines so as to better understand the mechanisms conferring radioresistance to these cells. We have determined the repercussions of IR on the expression of DNA damage response genes and DNA repair pathways.

## Methods

### GSC Cell lines, H9-NSC and Cell culture

Tumor samples were obtained within 30 min after surgical resection from 10 adult GBM patients (GSC-1, GSC-2, GSC-3, GSC-5, GSC-6, GSC-9, GSC-10, GSC-11, GSC-13 and GSC-14). The methodology for isolation and characterisation of these cells has been previously described [11, 19, 21]. All GSC lines were assessed for self-renewal, differentiation and in vitro clonogenicity by limiting dilution assays. In addition, tumorigenicity and stemness properties of GBM-derived stem cells were evaluated by xenograft experiments in nude mice [11, 19, 21]. Cells derived from all 10 tumors were cultured as proliferative non-adherent spheres in Neurobasal medium (NBE, Life Technologies, Carlsbad, CA, USA) supplemented with 20 ng/ml of basic fibroblast growth factor (bFGF, Life Technologies), 20 ng/ml of epidermal growth factor (EGF, Life Technologies) and culture supplements N2 (100X, Life Technologies) and B27 (50X, Life Technologies). Cells were used below passage 18 to 28. The molecular characteristics including MGMT promoter methylation, EGFR copy number, IDH1, IDH2, EGFR-variant III, p53, PTEN status as well as LOH at loci 1p36, 19q13, 9p21 and 10q23 of GSCs are indicated in Additional file 1: Table S2.

GIBCO® Human Neural Stem Cells (H9-NSCs) are derived from NIH-approved H9 (WA09) human embryonic stem cells. Cells were cultured following the manufacturer’s instructions.

### Cell irradiation

Gamma irradiation was performed at the Department of Radiotherapy (University Hospital of Poitiers) with an Elekta Synergy Beam Modulator (dose rate, 4.56Gy/min). Cells were kept on ice after IR and cultured at 37 °C. Control cells were subjected to the same experimental conditions.

### RI-1 treatment

Twenty-four hours before IR, GSCs were incubated with 10 μM of RAD-51 inhibitor RI-1 diluted in DMSO (Calbiochem, Nottingham, United Kingdom). RI-1 inhibitor covalently binds to RAD51 at Cys319, inhibiting subsequent recombinase activity [25, 26].

### Single cell gel electrophoresis (alkaline comet assay)

The comet assay was performed using the CometAssay kit (Trevigen, Gaithersburg, MD, USA) following the manufacturer’s instructions. Briefly, GSCs were enzymatically dissociated, 10^5^cells/mL were embedded in molten LMAgarose (0.5 % low-melting agarose) and incubated at 37 °C for 12 h before IR. At an indicated time after IR, the slides were transferred to lysis solution (Trevigen). A denaturation step was performed in alkaline solution (10 mM NaOH, 1 mM EDTA) at room temperature for 30 min. Electrophoresis was performed for 30 min at 25 V (300 mA) in an electrophoresis buffer (200 mM NaOH, 1 mM EDTA). The ethanol-fixed and dried slides were stained with SYBR Green (0.1 μL/ml; Exλ 488 nm, Emλ 520 nm). DNA breaks were analyzed in 100 cells using an image analysis system (Comet Imager, MetaSystems, Altlussheim, Germany). Olive Tail Moment (OTM) as a product of the tail length and the percentage of total DNA in the tail was applied to evaluate DNA breaks. Comet images were captured with the Axio Imager M2 fluorescent microscope (Carl Zeiss) at 20×.

### Cell proliferation: MTS assay

The effect of IR on doubling times of GSCs was assessed by CellTiter96®Aqueous Non-Radioactive Cell Proliferation Assay (Promega, Lyon, France). Cells were plated in 96-well plates at a density of 5 × 10^4^ cells per well in 100 μL medium. After 24 h of incubation, cells were irradiated at 4Gy or 12Gy. Quantification of viable cells was performed at 492 nm with a micro-plate reader (Dynex Technologies, Chantilly, France). The IC_50_ value was calculated as the drug concentration required to inhibit cell proliferation by 50 % compared with untreated control cells.

### Western blot analysis

10^6^ cells were irradiated at 4Gy and 12Gy. Cells were lyzed 45 min and 24 h after IR treatment in Laemmli buffer. Equal amounts of protein samples were separated by SDS-PAGE and transferred onto a nitrocellulose membrane (BioRad, Marnes-La-Coquette, France). Membranes were blocked with 5 % non-fat milk, 5 % BSA in PBS 0.1 % Tween and incubated overnight at 4 °C with RAD51 (SantaCruz, Texas, USA) and β-Actin (Abcam, Cambridge, UK) primary antibodies. After incubation with appropriate secondary antibodies (Cell signaling, Danvers, MA, USA), blots were revealed by chemiluminescence (BioRad). Band intensity was quantified using ImageJ software (Bethesda, MD, USA).

#### Analysis of gliomasphere mRNA by Low Density Array

TaqMan® Low Density Array (TLDA) was used (Life Technologies, Carlsbad, CA) to examine the expression of 46 human DNA repair genes in 10 GSCs before and 3 h following 4Gy. The list of target genes is detailed in Additional file 2: Table S1. Two microgram of total RNA were reverse transcribed using the High Capacity RNA-to-cDNA Kit according to the manufacturer’s instructions (Life Technologies). Real-time PCR experiments were then carried out with the ABI PRISM 7900HT Sequence Detection System. Each experiment was conducted in triplicate. Relative quantification (RQ) of target gene expression was determined by the 2^−ΔΔCt^ method using *glyceraldehyde 3-phosphate dehydrogenase* (*GAPDH*, most stable reference gene) as an endogenous control. Data were analyzed using the StatMiner 3.0 software (Integromics, Madrid, Spain).

#### RAD51 foci immunochemistry

Cells were treated with RI-1 during 24 h before IR and fixed with 4 % paraformaldehyde at the indicated times (45 min and 24 h after 12Gy IR). Cells were then blocked in PBS with 20 % donkey serum, 3 % BSA and 0.1 % Triton X-100, and stained with anti-RAD51 antibody (1:1000) (Euromedex, Souffelweyersheim, France) in blocking solution followed by Alexa488-conjugated secondary antibody (Life technologies). Nuclei containing more than five RAD51 foci were quantified by fluorescence microscopy in at least 100 cells (Axio Imager M2 fluorescent microscope, Carl Zeiss).

#### Annexin V and flow cytometry

GSCs were seeded 24 h before treatment with 10 μM RI-1 and/or 16Gy. After 7 days, cells were stained with Annexin V and 7AAD using a FITC Annexin V apoptosis detection kit (BD Biosciences, San Diego, CA, USA) following the manufacturer’s instructions and previous studies [11, 20]. Apoptosis was measured immediately by flow cytometry on a FACS Canto II (BD Biosciences). Data analysis was performed using FACS Diva software (BD Biosciences). A total of 10 000 events were analyzed in two independent experiments.

#### Tissue Microarray (TMA) construction, immunochemistry and scoring of RAD51 staining

TMAs were constructed using formalin-fixed paraffin embedded tissue samples that represent a total of 69 GBMs from surgical resection or biopsy patients operated in Poitiers University Hospital. Patient characteristics are summarized in Additional file 3: Table S3. All of these patients were treated with radiotherapy and temozolomide. Original slides were reviewed to confirm GBM histology according to the 2007 World Health Organization classification system. For each case, a minimum of 3 cores were transferred from the selected areas to the recipient block, using a TMA workstation (Alphelys, Plaisir, France). The recipient block was cut into 6 μm thick section, and immunochemistry was performed with an automated system (BenchMark XT, Ventana, Roche). Briefly, slides were deparaffinized and heated in Tris/Borate/EDTA pH8 solution for antigenic retrieval. The primary antibody RAD51 (Abcam, 1/50, 1h30) was incubated during 1h30 at 37 °C and revealed using the streptavidin-biotin-peroxidase method with diaminobenzidine as chromogen (UltraView universal DAB detection kit, Roche). Scoring of antibody staining was evaluated independently by two pathologists in a blind manner. Nuclear staining of RAD51 was scored as positive (more than one cell was stained) or negative. In case of interobserver variability, the slides were rescored by both pathologists until a consensus was reached.

#### Statistical analysis

With the exception of TLDA data (StatMiner), descriptive statistics of the results were calculated in GraphPad Prism 5 (La Jolla, CA, USA) or XLStat (Addinsoft, Paris, France). All experiments were performed at least three times. The results are presented as means ± standard deviation (SD), and statistical significance was evaluated by Mann Whitney and Student’s *t*-test (*p < 0.05, **p < 0.01). *p* values less than 0.05 were considered statistically significant. Log-rank analysis was applied to Kaplan-Meier survival curves.

## Results

### DNA repair kinetics following IR exposure in glioblastoma stem cells

**Fig. 1:**
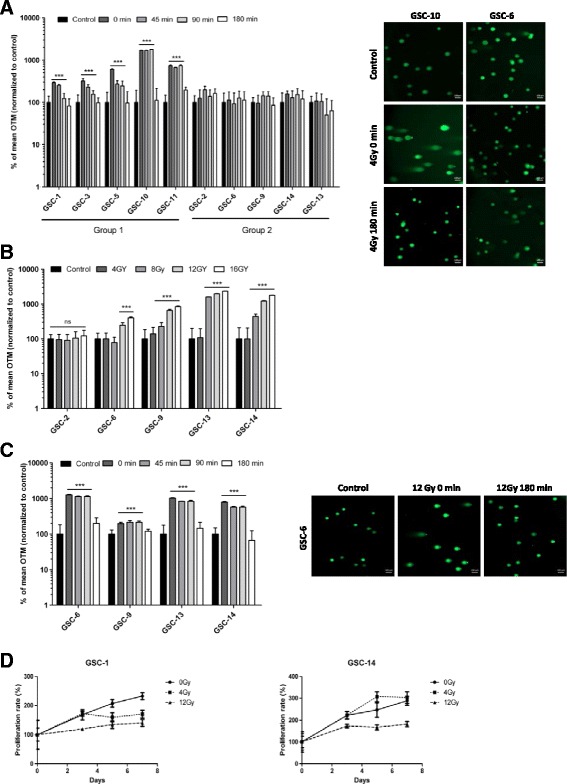
Measurement of DNA damage and cell proliferation in GSCs following IR. **a** 10 GSC lines were irradiated at 4Gy and subjected to comet assay at the indicated time. Data are given as a percentage of olive tail moment (OTM) and normalized to control (***p < 0.001 versus control cells). **b** GSCs from group 2 were irradiated at the indicated doses and subjected to comet assay immediately thereafter. Data are given as a percentage of olive tail moment (OTM) and normalized to control (***p < 0.001 versus control cells). **c** GSCs from group 2 were irradiated at 12Gy and subjected to comet assay at the indicated time. Data are given as a percentage of olive tail moment (OTM) and normalized to control (***p < 0.001 versus control cells). **d** Cell proliferation was measured 7 days after IR (4Gy and 12Gy) using an MTS assay (T0 = IR). Each set of results was obtained from three independent experiments. Experiments were performed in sextuplicate and expressed as mean ± SD. Doubling times were extrapolated based on exponential growth equations

To investigate the kinetics of DNA repair in glioblastoma stem cells after IR, we conducted a study on a series of 10 GSCs. Cells were exposed to 4Gy IR and DNA damage was monitored by single-cell gel electrophoresis or “comet assay” in alkaline conditions so as to simultaneously detect both double and single-strand DNA breaks with high sensitivity [22]. Levels of DNA damage were expressed as mean OTM (±SD) and normalized to untreated control cells; an increase in Olive Tail Moment (OTM) reflected an increase of DNA breaks in cells. Our results revealed heterogeneous DNA repair kinetics at 4Gy (Fig. 1a). Immediately after IR (t = 0 min), a marked increase in DNA damage (as much as 2- to 17-fold) was seen in GSC-1, -3, -5, -10, -11 (*p* < 0.001). Analysis of later time points (45, 90 and 180 min) revealed that the majority of DNA breaks were resolved by 180 min, with a return to basal level. In other GSC lines -2, -6, -9, -14, and -13, no significant accumulation of DNA damage was observed after 4Gy IR during the same time-course. Representative images of comet assays are shown in Fig. 1a. This series of GSCs may be divided into two groups according to their radiosensitivities at 4Gy, a radiosensitive group (group 1) including GSC-1, -3, -5, -10, and -11, and a radioresistant group (group 2) including GSC-2, -6, -9, -14, and -13. To induce DNA damage in group 2, cells were exposed to increased radiation doses and DNA breaks were monitored immediately thereafter (t = 0 min) (Fig. 1b). As previously observed, no damage could be detected after exposure to 4Gy. OTM significantly increased following exposure to 8Gy in GSC-9, -13 and -14, and to 12Gy in GSC-6 (*p* < 0.001). A particularly noteworthy observation was made for GSC-2 since no damage could be detected at any dose tested (up to 16Gy), hence this cell line seemed to be highly resistant to IR.

In addition, we performed comet assay in H9-derived Human Neural Stem Cells (H9-NSC) to explore their DNA damage response after IR. Immediately after 4Gy IR (t = 0 min) OTM significantly increased (*p* < 0.001) in H9-NSC but remained elevated up to 180 min (*p* < 0.001) (Additional file 4: Figure S1A).

To further evaluate DNA repair kinetics in cells from group 2, we performed comet assay after 12Gy in GSC-6, -9, -13 and -14. Promptly upon IR (t = 0 min), OTM significantly increased up to 10-fold (*p* < 0.001) (Fig. 1c). Within 180 min after exposure to IR, OTM decreased and returned to basal levels in all four GSCs tested, indicating that cells were able to resolve DNA breaks following a high dose of IR. Representative images from comet assays are shown in Fig. 1c.

We next determined the effects of IR on cell proliferation using an MTS assay in two GSC lines from group 1 (GSC-1) and group 2 (GSC-14) (Fig. 1d). Doubling times of GSC-1 and GSC-14 were 5.7- and 4.7-days respectively. As expected, exposure to 4Gy IR decreased the proliferation rate of GSC-1 (9.6-days), whereas no similar effect was observed on GSC-14 (4.3-days). A higher dose of IR (12Gy) decreased the proliferation rates of GSC-1 and GSC-14 and increased doubling times to 14.1- and 8.7-days respectively (Fig. 1d). These observations were consistent with results obtained from comet assay.

### RAD51 expression and radioresistance of GSCs

**Fig. 2:**
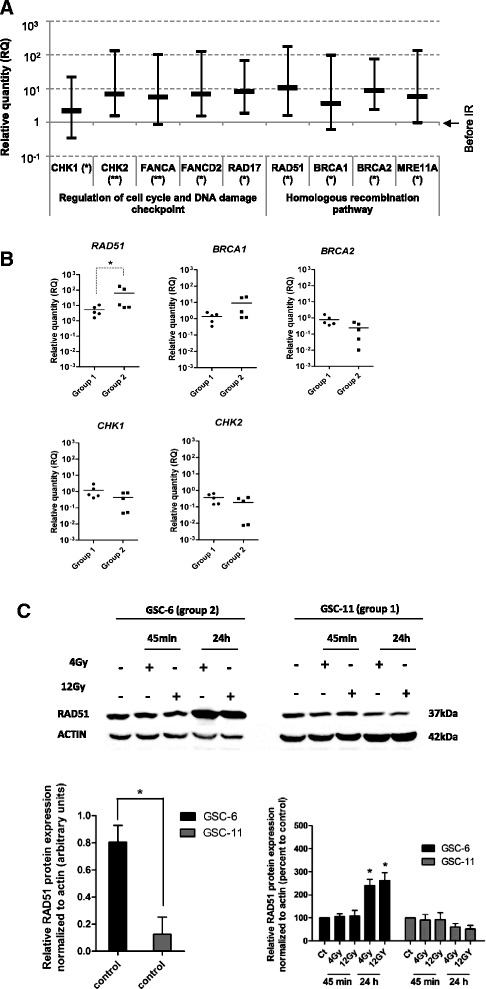
Expression of DNA repair genes in GSCs after IR. **a** TLDA expression levels of the most significant DNA repair genes. Relative expressions were measured 3 h following IR, data represent the mean ± SD of 10 GSCs determined by 2^-ΔΔCt^ quantification method. Relative expressions of target genes were determined using *GAPDH* as endogenous control (**p < 0.01, *p < 0.05). **b** mRNA expression of *RAD51*, *BRCA1*, *BRCA2*, *CHK1* and *CHK2* in group 1 and group 2. The vertical scatter plot shows the log10 expression of relative quantification (RQ) values normalized to the expression before IR. Each data point represents one GSC line measured in triplicate (*p = 0.032). **c** Western blot analysis of RAD51 following 4Gy and 12Gy IR. Total protein were extracted after 45 min and 24 h following IR, β-actin was used as loading control. Densitometric analysis of specific signals shows relative RAD51 protein expression levels normalized with β-actin and expressed as a percentage of control in GSC-6 and GSC-11 (*n* = 3) (*p < 0.05) (Image J software)

To monitor the DNA repair processes triggered by IR in GSCs, we designed custom Taqman Low Density Array (TLDA) for genes involved in HR, NHEJ, BER (Base Excision Repair), NER (Nucleotide Excision Repair), DNA damage sensing and cell cycle control (Additional file 2: Table S1). After exposure to 4Gy, RNA levels of critical DNA damage response genes increased (Fig. 2a). For all the GSCs tested, exposure to IR increased *CHK1*, *CHK2* (*Checkpoint Kinases 1 and 2*) and *RAD17* levels. Chk1 and Chk2 kinases are known to play a critical role in cellular responses to DNA damage by initiating cell cycle arrest in GSCs [4]. RAD17 was shown to be a key regulator of the cell cycle checkpoint [23]. We also observed increased *FANCA* and *FANCD2 (Fanconi Anemia complementation group A and D2)* expression after IR; both genes being required for intra-S-phase checkpoint [24]. Effectors of HR such as *BRCA1 (Breast Cancer 1)*, *BRCA2*, *MRE11A* and *RAD51* were significantly expressed following IR. We then focused on genes differentially expressed between the two groups of cells (Additional file 2: Table S1). Of note, only *RAD51* expression showed a significant difference between the two groups of GSCs (*p* = 0.032). RAD51 was highly expressed after exposure to 4Gy IR in group 2 compared to group 1 (Fig. 2b). No significant difference in expression was found for other genes involved in HR, such as *BRCA1*, *BRCA2*, *CHK1* and *CHK2*. (Additional file 2: Table S1). RAD51 expression was lower in H9-NSC (*p* < 0.05) as compared with the two groups of GSCs (Additional file 4: Figure S1B).

**Fig. 3:**
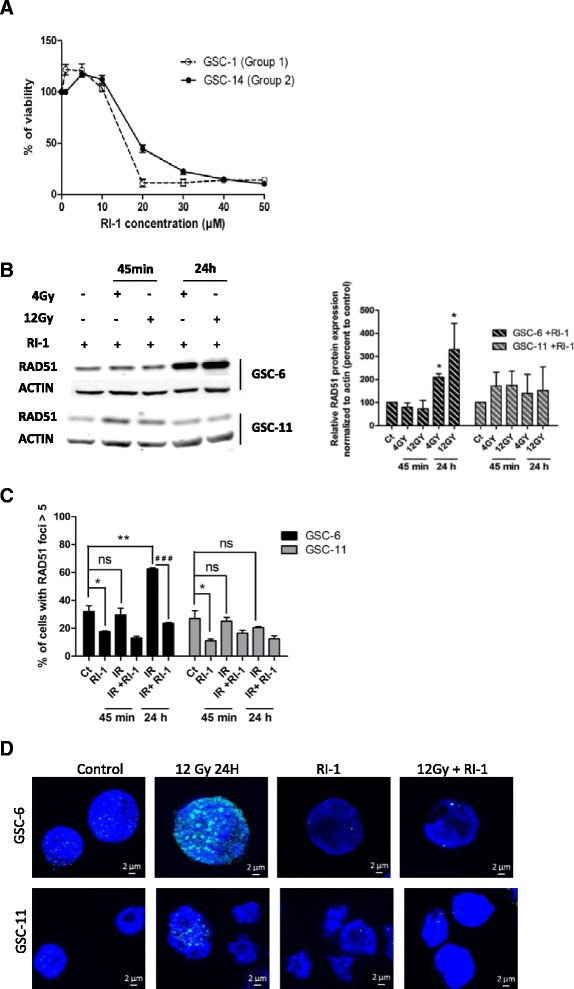
Chemical inhibitor of RAD51, RI-1, inhibits RAD51 foci formation. **a** GSCs viability was measured using an MTS assay after 5 days of RI-1 treatment. IC_50_ values were 22.3 μM and 19.7 μM respectively for GSC-14 and GSC-1. **b** Western blot analysis of RAD51 was performed on GSCs treated for 24 h with 10 μM RI-1 before IR. Total protein samples were extracted after 45 min and 24 h following 4Gy and 12Gy IR. β-actin was used as a loading control. Densitometric analysis of specific signals shows relative RAD51 protein expression levels normalized with β-actin and expressed as a percentage of control in GSC-6 and GSC-11 (*n* = 3) (*p < 0.05) (Image J software). **c** Cells were treated for 24 h with 10 μM of RI-1 before 12Gy IR and harvested at the indicated times. For each time point, the number of cells with RAD51 foci > 5 was scored and expressed as a percentage of the total number of nucleus scored. (*p < 0.05, **p < 0.01, ### *p* < 0.001, ns = no significant). **d** Representative images of GSC-6 and GSC-11 treated with RI-1. RAD51 foci (*green*) and nucleus (*blue*) are shown after 24 h following 12Gy exposure. These images were captured with the Axio Imager M2 fluorescent microscope (Carl Zeiss), scale bar: 2 μm

In a manner consistent with data obtained from mRNA analysis, western blot analysis revealed significantly higher levels of RAD51 protein before IR in GSC-6 (group 2) compared with GSC-11 (group 1) (*p* < 0.05) before IR (Fig. 2c). RAD51 protein expression increased after 24 h following 4Gy and 12Gy exposure in GSC-6 (group 2) compared with control cells (*p* < 0.05). By contrast, in GSC-11, RAD51 protein levels remained unchanged after 45 min and 24 h following IR (Fig. 2c). Moreover, immunofluorescence staining revealed a significant increase of RAD51 foci-positive cells (>5 foci per nucleus) after 24 h following 12Gy IR in GSC-6 compared with control cells (*p* < 0.01) (Fig. 3c). In GSC-11, the percentage of RAD51 foci-positive cells remained unchanged after 45 min and 24 h following IR (no statistically significant difference) (Fig. 3c). Taken together, these findings indicate that RAD51 expression is differentially expressed between the two groups of GSCs following IR, suggesting its potential role in radioresistance.

### Effects of RAD51 inhibition on GSCs after IR

To evaluate the contribution of RAD51 in the radioresistance of GSCs from group 2, we inhibited RAD51 with a chemical inhibitor, RI-1, which irreversibly destabilizes the formation of RAD51 filaments [25, 26]. RI-1 inhibitor dramatically decreased cell viability of GSC-1 (group 1) and GSC-14 (group 2) at a concentration of 20 μM to 50 μM (Fig. 3a). In our experimental design, we used 10 μM of RI-1 without further effect on cell viability. This dose of inhibitor was consistent with previous studies performed on leukemic cells (15 μM) [27] and fibroblasts (10 μM) [28]. Likewise, RI-1 inhibitor had no effect on cell viability of H9-NSC up to 15 μM. (Additional file 4: Figure S1C).

We then determined the impact of RI-1 inhibitor on RAD51 protein expression in GSC-6 and GSC-11. Cells were treated for 24 h with RI-1 and RAD51 levels measured at 45 min and 24 h following 4Gy and 12Gy IR exposure (Fig. 3b). RAD51 protein expression significantly increased in GSC-6 after 24 h (*p < 0.05) but remained unchanged in GSC-11 (Fig. 3b), indicating that RI-1 inhibitor had no measurable effect on RAD51 protein expression. This observation is in line with previous reports indicating a covalent binding of RI-1 inhibitor to the RAD51 surface, destabilizing filament formation and preventing DNA damage repair without altering protein expression [25, 26]. To assess RI-1 inhibition in GSCs we analyzed RAD51 foci formation before and after RI-1 treatment. In the absence of IR, RI-1 treatment significantly decreased the number of RAD51 foci-positive cells in both GSC-6 and GSC-11 (*p* < 0.05) (Fig. 3c). RI-1 treatment prevented foci formation in GSC-6 cells following 12Gy IR as a significant reduction in the percentage of foci-positive cells was observed after 24 h in RI-1 treated cells (*p* < 0.001) (Fig. 3c). Representative images from immunofluorescence staining are shown in Fig. 3d.

**Fig. 4:**
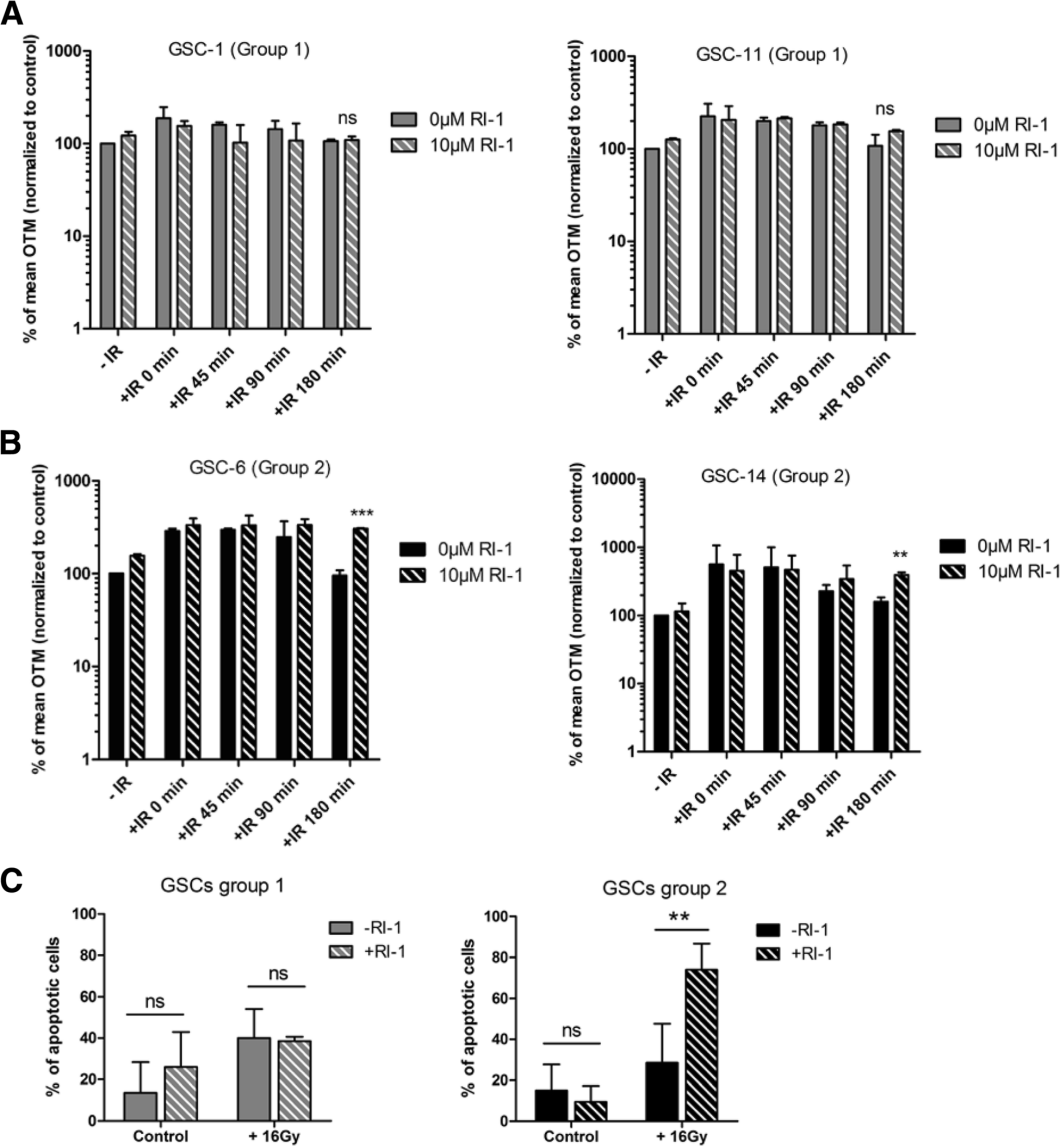
Chemical inhibitior of RAD51, RI-1, selectively radiosensitizes GSCs from group 2. Comet assay was performed on GSCs from group 1 (GSC-1 and - 11) (**a**) and group 2 (GSC-6 and -14) (**b**) treated for 24 h with 10 μM RI-1 before undergoing 16Gy IR. Data are given as a percentage of olive tail moment (OTM) normalized to control (***p < 0.001, **p < 0.01 versus control cells, ns = no significant). **c** Apoptosis was measured in both groups 7 days after treatment with 10 μM RI-1 and IR. Annexin V/7-AAD labeling was analyzed by flow cytometry (**p < 0.01, ns = no significant)

Cells from group 1 (GSC-1 and -11) and group 2 (GSC-6 and-14) were treated with 10 μM of RI-1 during 24 h and irradiated with 16Gy before performing an alkaline comet assay. In unirradiated GSCs, OTM were not affected following inhibition of RAD51 (Fig. 4a, b). Kinetic analysis of DNA repair in irradiated cells from group 1 (GSC-1 and -11) did not show significant modification of OTM following RI-1 treatment, as measured up to 180 min (Fig. 4a). In contrast, significant increases in OTM were observed after 180 min in GSCs from group 2 (GSC-6 and -14) (*p* < 0.001) in the presence of RI-1 (Fig. 4b). From these results, inhibition of RAD51 appears to radiosensitize GSCs from group 2. Representative images of comet assays are shown in Additional file 5: Figure S2A and S2B. Similar experiments were conducted using 4Gy IR doses; however, only a minor effect was observed on OTM (data not shown). H9-NSC were treated with 10 μM of RI-1 during 24 h and irradiated with only 4Gy as these cells are very sensitive to IR. OTM were not significantly modified following RI-1 when compared with untreated cells. (Additional file 4: Figure S1D).

Previous studies have demonstrated that IR induce activation of apoptosis in glioblastoma cell lines and that targeting of DNA damage response radiosensitizes cells by enhancing apoptosis [29–31]. To analyze the effects of RAD51 inhibitor on GSC apoptosis, we performed Annexin V staining of GSCs after exposure to 16Gy. RI-1 treatment did not affect the apoptosis rate in unirradiated cells as measured after 7 days in both groups (Fig. 4c). Combination of RI-1 treatment and 16Gy significantly increased (*p* = 0.004) the fraction of apoptotic cells in group 2 and the amount of apoptotic cells reached 74 % in comparison with 28.5 % for IR alone (Fig. 4c). Unlike group 2, this combination therapy did not enhance the apoptotic index of cells from group 1 (38.5 vs. 40 %) (Fig. 4c).

### Patients outcome and radiosensitivity of GSC

In an attempt to extrapolate the clinical consequences of our previous in vitro observations, we addressed the question whether patients from group 1 and group 2 (i.e. related to GSC group 1 and 2 respectively) may have a different outcome. Interestingly, in line with our in vitro results, comparison of progression-free survival (PFS) in patients of group 1 (low basal expression of RAD51 in GSCs) and group 2 (high basal expression of RAD51 in GSCs) revealed a better outcome for patients of group 1 (PFS ≥6 months in group 1 and PFS <6 months in group 2) (Additional file 3: Table S3 and Additional file 6: Figure S3), suggesting a potential involvement of RAD51 in tumor radioresistance.

### Patient outcome and RAD51 protein expression in tumors

**Fig. 5:**
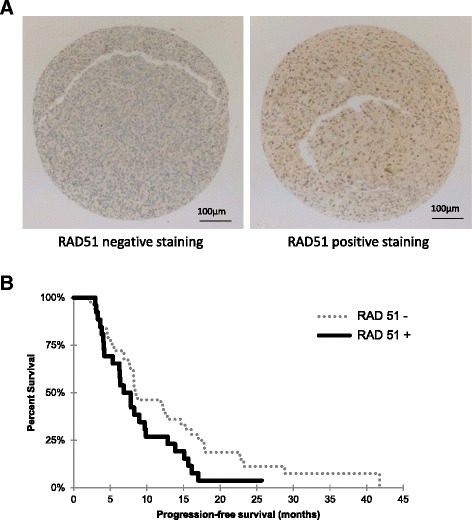
RAD51 protein expression in GBM tumors is associated with shorter progression-free survival. **a** Representative sections of TMA stained with RAD51 were analyzed by immunohistochemistry. All images were obtained at magnification 4× (scale bar 100 μm). The *left section* showed no RAD51 staining and the right section showed RAD51 staining. **b** Kaplan-Meier curve of all glioblastoma patients plotting progression-free survival for patients with low or high expression of RAD51 protein (*p* = 0.065)

Considering the architectural heterogeneity and the small number of cancer stem cells that indeed exist within GBMs, this result should be considered with caution. To accumulate further evidence for a potential role of RAD51 in tumor radioresistance, we constructed a TMA including 69 patients with resected GBM. RAD 51 protein expression was observed in 36 % of the samples (Table 1), Fig. 5a shows a representative spot of RAD51 positive and negative staining (RAD51 +, RAD51 -). The PFS tended to be higher in patients without RAD51 staining (8.5 months) compared with patients having a detectable RAD51 expression (6.8 months), but these results failed to achieve statistical significance (*p* = 0.065) (Fig. 5b). In addition, patients with higher RAD51 expression had poorer mean overall survival (14.1 vs. 18.5 months) although this difference was still not significant (data not shown).

**Table 1:** RAD51 protein expression in GBM tumors. *M* Male, *F* Female, *OS* Overall survival, *PFS* Progression-free survival

	Number of case	Median age (range)	Gender		Median PFS (range; months)	OS median (range; months)
			F	M		
RAD51 +	26	63 (39–76)	10	16	6.8 (2.9–25.6)	14.1 (5.3–70.1)
RAD51 -	43	60 (32–83)	11	32	8.5 (2–41.8)	18.5 (3.1–45.4)

## Discussion

Current treatment for GBM includes surgical resection followed by concomitant chemotherapy and radiotherapy. Despite the extent of resection, residual radioresistant GSCs continue to propagate after radiotherapy leading to tumor recurrences [32, 33]. In this study, we used single cell gel assay (comet assay) to quantify DNA damage and measure DNA repair post-irradiation in 10 GBM-derived cell lines. Our results have underscored wide differences in the radiosensitivity of GSCs derived from tumors of the same histology, highlighting two distinct groups. The first group (1) has been characterized including GSCs showing high levels of DNA damage following 4Gy IR, and the second group (2) with increased radioresistance (up to 16Gy) showing undamaged DNA after 4Gy IR. Hence, these results demonstrate the heterogeneity of GSC response to radiation with the existence of different thresholds for triggering DNA damage response and repair. Interestingly, all the GSCs tested displayed functional and efficient DNA repair machinery as evidenced by fast repair kinetics.

Previous experiments by Lim et al. [8, 17] highlighted the preferential activation of HR pathway in GSCs following DNA damage induced by IR. Our data for mRNA expression corroborate this previous study, through the analysis of 46 DNA repair genes post-irradiation. We observed increased expression of genes involved in HR pathway and cell cycle regulation like *CHK1*, *CHK2, BRCA1, BRCA2* and *RAD51*. CHK1 and CHK2 are known to be involved in cell cycle arrest and extended DNA repair capacity of GSCs [4, 34]. In response to single strand breaks, CHK1 activation leads to S and G2/M arrest while CHK2 activation induces G1 arrest in response to double strand breaks [35]. BRCA1 and BRCA2 are relocated with RAD51 to sites of DNA damage and replication forks. RAD51 is loaded onto DNA breaks to form a nucleoprotein filament mediating HR followed by replication using neighboring undamaged sister chromatids [36].

Several studies have demonstrated that GSCs preferentially activate the HR pathways to repair DNA damage [8, 17, 37], and inhibition of HR increased the sensitivity of GSCs to IR [17]. Analysis of gene expression after IR revealed differential mRNA and protein expression for RAD51 between the two groups with higher levels in group 2. RAD51 levels have been shown to be higher in cancer cells as compared to normal cells [38]. Moreover, in prostate and breast cancer, high RAD51 protein levels appear to be consistent with tumor progression and resistance to therapy [39, 40]. In this study we focused on 69 patients with GBM exclusively treated with temozolomide and radiotherapy, there was no significant difference in the PFS according to RAD51 protein expression although the PFS of patients with high RAD51 expression was shorter. Further studies have demonstrated the involvement of RAD51 in resistance to IR in several established human glioma cell lines with RAD51 inhibition enhancing radiosensitivity of these cells [18, 41, 42]. In an attempt to increase the radiosensitivity of GSCs from group 2, we inhibited RAD51 activity by RI-1 treatment. Previous authors have used RI-1 to inhibit RAD51 and to enhance the sensitivity to IR in leukemic T-cells [27]. This strategy increased IR efficacy through specific inhibition of HR, enhancing DNA damage and causing death in these cells. In the present study, inhibition of RAD51 by RI-1 treatment significantly increased DNA damage and apoptosis post-irradiation (16Gy) in GSCs from group 2 expressing high RAD51 levels. However we did not observe an effect of RI-1 on H9-NSC (neural stem cells) following DNA damage.

These results identified RAD51 as a promising target helping to selectively radiosensitize subgroups of GBM and confirmed the importance of RAD51 in the radioresistance mechanisms of GSCs. In line with our findings Konstantinopoulos et al., found that suberoylanilide hydroxamic acid, known to downregulate RAD51 in combination with olaparib, a PARP (polyADP-ribose polymerase) inhibitor, significantly decreased the viability of HR-proficient and -deficient ovarian cancer cell lines [43]. In addition, RAD51 small molecule inhibitors are currently being developed for cancer clinical trials [44–46]. Notwithstanding the beneficial effect observed after RAD51 inhibition in eradication of GSCs, several studies have demonstrated that GSCs reside predominantly in highly hypoxic or anoxic areas in vivo in a quiescent and nonproliferative state, with a considerably reduced response to radiotherapy [47, 48]. Hence in addition to this in vitro study further in vivo studies combining orthotopic xenografts of GSCs in animal models and RAD51 inhibition will be necessary in view of developing more effective therapeutic strategies.

## Conclusions

Taken together, our data have confirmed the importance of RAD51 in the radioresistance mechanisms of GCSs and inhibition of RAD51 resulted in decreased DNA repair leading to cell death in GSCs expressing high RAD51 levels. Consequently, the inhibition of RAD51 and HR pathways could be an effective adjuvant to the current standard treatment of GBM and represent a major advance for difficult-to-treat cancers.

## Abbreviations

BER, base excision repair; BRCA1/2: breast cancer 1/2; CHK, checkpoint kinase; DSB, double-strand breaks; FANCA/FANCD2, Fanconi Anemia complementation group A/ group D2; GBM, glioblastoma; GSC, glioblastoma stem cells; HR, homologous recombination; IR, ionizing radiations; NER, nucleotide excision repair; NHEJ, non-homologous end joining; OTM, olive tail moment; PFS, progression-free survival; STAT3, signal transducer and activator of transcription 3; TLDA, taqman low density array

## Additional files

Additional file 1: Table S2. Analysis of ten glioblastoma-derived GSCs. LOH, Loss Of Heterozygosity; IDH, Isocitrate Dehydrogenase; WT, Wildtype; Mut, Mutant; PTEN, Phosphatase and TENsin homolog; EGFR, Epidermal Growth Factor Receptor; MGMT, O-6-methylguanine-DNA methyltransferase; PFS, Progression-free survival, IQ, insufficient quantity. Group 1 and group 2 are described in the results section. (DOCX 16 kb) Additional file 2: Table S1. Characteristics of the DNA Repair TLDA and *p* values of expression ratio between group 1 and group 2 after IR*.* The microfluidic card assays included 46 DNA repair genes and 2 housekeeping genes. To validate these assays, accuracy and reproducibility of amplification data were evaluated on triplicated samples. *p* values comparing expression of group 1 versus group 2 after IR were determined by using StatMiner software. RAD51 was significantly differentially expressed between the two groups (**p* < 0.05). GSCs of group 1 and group 2 are described in the results section. (DOCX 16 kb) Additional file 3: Table S3. Patients and tumors characteristics. M, Male; F, Female; OS, Overall survival; PFS, Progression-free survival. (DOCX 13 kb) Additional file 4: Figure S1. Measurement of DNA damage and RAD51 expression in H9-NSC cells. A) H9-NSC line was irradiated at 4Gy and subjected to comet assay at indicated time. Data are given as a percentage of olive tail moment (OTM) and normalized to control (****p* < 0.001 versus control cells). B) mRNA expression of RAD51 in group 1, group 2 and H9-NSC (**p* < 0.05). The vertical scatter plot shows the log10 expression of relative quantification (RQ) values normalized to the expression before IR. Each data point represents each cell line measured in triplicate. C) H9-NSC viability was measured using an MTS assay after 5 days of RI-1 treatment. D) Comet assay was performed on H9-NSC treated for 24 h with 10 μM of RI-1 before 4Gy IR. Data are given as a percentage of olive tail moment (OTM) normalized to control. (PPTX 326 kb) Additional file 5: Figure S2. Representative comet images of A) GSC-11 (group 1) and B) GSC-14 (group 2) after RI-1 treatment and 16Gy IR (t = 180 min) Comet images were captured with the Axio Imager M2 fluorescent microscope (Carl Zeiss) at 20× (scale bar 100 μm). IR, Ionizing radiation (PPTX 280 kb) Additional file 6: Figure S3. Relation between patients outcome and radiosensitivity of GSCs. GSCs from group 1 and group 2 are described in the results section; PFS, Progression-free survival. (PPTX 94 kb)
