# Supplementary material for: A radiosensitizing effect of RAD51 inhibition in glioblastoma stem-like cells
Source: BMC Cancer. 2016 Aug 5;16:604. doi: 10.1186/s12885-016-2647-9 (PMC4974671; doi:10.1186/s12885-016-2647-9)
Supplement: Additional file 1: Table S2. — Analysis of ten glioblastoma-derived GSCs. LOH, Loss Of Heterozygosity; IDH, Isocitrate Dehydrogenase; WT, Wildtype; Mut, Mutant; PTEN, Phosphatase and TENsin homolog; EGFR, Epidermal Growth Factor Receptor; MGMT, O-6-methylguanine-DNA methyltransferase; PFS, Progression-free survival, IQ, insufficient quantity. Group 1 and group 2 are described in the results section. (DOCX 16 kb) [file 12885_2016_2647_MOESM1_ESM.docx]

|  | **GSC-1** | **GSC-3** | **GSC-5** | **GSC-10** | **GSC-11** | **GSC-2** | **GSC-6** | **GSC-9** | **GSC-13** | **GSC-14** |
| --- | --- | --- | --- | --- | --- | --- | --- | --- | --- | --- |
|  | **Group 1** | **Group 1** | **Group 1** | **Group 1** | **Group 1** | **Group 2** | **Group 2** | **Group 2** | **Group 2** | **Group 2** |
| LOH 1p | negative | negative | negative | negative | negative | negative | negative | negative | negative | positive |
| LOH 19q | negative | positive | negative | positive | negative | negative | negative | negative | negative | positive |
| LOH 9p21 | negative | positive | positive | positive | IQ | positive | positive | positive | positive | positive |
| LOH 10q | positive | positive | positive | positive | IQ | positive | positive | positive | positive | positive |
| IDH1 status | WT | WT | WT | WT | WT | WT | WT | WT | WT | WT |
| IDH2 status | WT | WT | WT | WT | WT | WT | WT | WT | WT | WT |
| p53 status | WT | WT | Mut | WT | WT | WT | Mut | Mut | WT | WT |
| PTEN status | Mut | Mut | WT | Mut | WT | Mut | Mut | Mut | Mut | WT |
| EGFR VIII mutation | Mut | Mut | Mut | Mut | Mut | WT | Mut | Mut | Mut | Mut |
| EGFR Copy number | Polysomy | Amplification Trisomy  chr 7 | Duplication | Trisomy chr 7 | Polysomy | Polysomy | Trisomy chr 7 | Duplication | Amplification Trisomy  chr 7 | Trisomy chr 7 |
| MGMT promoter methylation | 4% | 7% | 2% | 87% | 94% | 2% | 34% | 51% | 1% | 19% |
| PFS | 8 months | 6 months | 6 months | 10 months | 10 months | 3 months | 2 months | 2 months | 5 months | 5 months |

**Table S2: Analysis of ten glioblastoma-derived GSCs**. LOH, Loss Of Heterozygosity; IDH, Isocitrate Dehydrogenase; WT, Wildtype; Mut, Mutant; PTEN, Phosphatase and TENsin homolog; EGFR, Epidermal Growth Factor Receptor; MGMT, O-6-methylguanine-DNA methyltransferase; PFS, Progression-free survival, IQ, insufficient quantity. Group 1 and group 2 are described in the results section.
