# Supplementary material for: A radiosensitizing effect of RAD51 inhibition in glioblastoma stem-like cells
Source: BMC Cancer. 2016 Aug 5;16:604. doi: 10.1186/s12885-016-2647-9 (PMC4974671; doi:10.1186/s12885-016-2647-9)
Supplement: Additional file 2: Table S1. — Characteristics of the DNA Repair TLDA and p values of expression ratio between group 1 and group 2 after IR. The microfluidic card assays included 46 DNA repair genes and 2 housekeeping genes. To validate these assays, accuracy and reproducibility of amplification data were evaluated on triplicated samples. p values comparing expression of group 1 versus group 2 after IR were determined by using StatMiner software. RAD51 was significantly differentially expressed between the two groups (*p < 0.05). GSCs of group 1 and group 2 are described in the results section. (DOCX 16 kb) [file 12885_2016_2647_MOESM2_ESM.docx]

| **Gene** | **Abbreviation** | **p values of ratio of group 1/ group 2 after IR** |
| --- | --- | --- |
| *APEX1* | APEX nuclease (multifunctional DNA repair enzyme) 1 | 0.22 |
| *ATM* | ataxia telangiectasia mutated | 0.27 |
| *ATR* | ataxia telangiectasia and Rad3 related | 0.23 |
| *BRCA1* | breast cancer 1, early onset | 0.11 |
| *BRCA2* | breast cancer 2, early onset | 0.37 |
| *CHEK1* | CHK1 checkpoint homolog (S. pombe) | 0.12 |
| *CHEK2* | CHK2 checkpoint homolog (S. pombe) | 0.17 |
| *ERCC1* | excision repair cross-complementing rodent repair deficiency, complementation group 1 (includes overlapping antisense sequence) | 0.81 |
| *ERCC2* | excision repair cross-complementing rodent repair deficiency, complementation group 2 | 0.23 |
| *ERCC3* | excision repair cross-complementing rodent repair deficiency, complementation group 3 (xeroderma pigmentosum group B complementing) | 0.27 |
| *ERCC4* | excision repair cross-complementing rodent repair deficiency, complementation group 4 | 0.29 |
| *ERCC5* | excision repair cross-complementing rodent repair deficiency, complementation group 5 | 0.26 |
| *ERCC6* | excision repair cross-complementing rodent repair deficiency, complementation group 6 | 0.34 |
| *ERCC8* | excision repair cross-complementing rodent repair deficiency, complementation group 8 | 0.48 |
| *FANCA* | Fanconi anemia, complementation group A | 0.19 |
| *FANCD2* | Fanconi anemia, complementation group D2 | 0.18 |
| *FEN1* | flap structure-specific endonuclease 1 | 0.13 |
| *GADD45A* | growth arrest and DNA-damage-inducible, alpha | 0.90 |
| *GTF2H1* | general transcription factor IIH, polypeptide 1, 62kDa | 0.17 |
| *GTF2H3* | general transcription factor IIH, polypeptide 3, 34kDa | 0.11 |
| *LIG1* | ligase I, DNA, ATP-dependent | 0.14 |
| *LIG3* | ligase III, DNA, ATP-dependent | 0.16 |
| *LIG4* | ligase IV, DNA, ATP-dependent | 0.26 |
| *MRE11A* | MRE11 meiotic recombination 11 homolog A (S. cerevisiae) | 0.22 |
| *NBN* | nibrin | 0.16 |
| *PARP1* | poly (ADP-ribose) polymerase 1 | 0.14 |
| *PCNA* | proliferating cell nuclear antigen | 0.31 |
| *POLA1* | polymerase (DNA directed), alpha 1, catalytic subunit | 0.24 |
| *POLB* | polymerase (DNA directed), beta | 0.08 |
| *POLD1* | polymerase (DNA directed), delta 1, catalytic subunit 125kDa | 0.21 |
| *POLH* | polymerase (DNA directed), eta | 0.40 |
| *POLK* | polymerase (DNA directed) kappa | 0.73 |
| *PRKDC* | protein kinase, DNA-activated, catalytic polypeptide | 0.07 |
| *RAD17* | RAD17 homolog (S. pombe) | 0.32 |
| *RAD18* | RAD18 homolog (S. cerevisiae) | 0.27 |
| *RAD50* | RAD50 homolog (S. cerevisiae) | 0.70 |
| *RAD51* | RAD51 homolog (S. cerevisiae) | **0.03 (*)** |
| *RAD52* | RAD52 homolog (S. cerevisiae) | 0.09 |
| *RPA2* | replication protein A2, 32kDa | 0.17 |
| *TP53* | tumor protein p53 | 0.44 |
| *XPA* | xeroderma pigmentosum, complementation group A | 0.21 |
| *XPC* | xeroderma pigmentosum, complementation group C | 0.60 |
| *XRCC1* | X-ray repair complementing defective repair in Chinese hamster cells 1 | 0.16 |
| *XRCC4* | X-ray repair complementing defective repair in Chinese hamster cells 4 | 0.66 |
| *XRCC5* | X-ray repair complementing defective repair in Chinese hamster cells 5 (double-strand-break rejoining) | 0.20 |
| *XRCC6* | X-ray repair complementing defective repair in Chinese hamster cells 6 | 0.33 |
|  |  |  |
| **Enogenous control** |  |  |
| *GAPDH* | glyceraldehyde-3-phosphate dehydrogenase |  |
| *18S* | Eukaryotic 18S rRNA |  |

**Table S1: Characteristics of the DNA Repair TLDA and p values of expression ratio between group 1 and group 2 after IR.** The microfluidic card assays included 46 DNA repair genes and 2 housekeeping genes. To validate these assays, accuracy and reproducibility of amplification data were evaluated on triplicated samples. p values comparing expression of group 1 *versus* group 2 after IR were determined by using StatMiner software. RAD51 was significantly differentially expressed between the two groups (*p<0.05). GSCs of group 1 and group 2 are described in the results section.
