# Supplementary material for: A radiosensitizing effect of RAD51 inhibition in glioblastoma stem-like cells
Source: BMC Cancer. 2016 Aug 5;16:604. doi: 10.1186/s12885-016-2647-9 (PMC4974671; doi:10.1186/s12885-016-2647-9)
Supplement: Additional file 3: Table S3. — Patients and tumors characteristics. M, Male; F, Female; OS, Overall survival; PFS, Progression-free survival. (DOCX 13 kb) [file 12885_2016_2647_MOESM3_ESM.docx]

|  | Glioblastomas (69) |
| --- | --- |
| *Gender :*  M  F | 48  21 |
| *Age at diagnosis* :  <60 years  ≥ 60 years | 31  38 |
| *median OS (months)* | 8.26 |
| *median PFS (months)* | 15.7 |
| *Surgery :*  Total excision  Partial excision  Biopsy | 38  12  15 |
| *Radiotherapy*  60Gy  40Gy | 65  4 |
| *Temozolomide*  Concomitant (+/- adjuvant)  Adjuvant alone  At recurrence  Neoadjuvant | 57  6  2  1 |

**Table S3: Patients and tumors characteristics.** M, Male; F, Female; OS, Overall survival; PFS, Progression-free survival.
