# Supplementary material for: A radiosensitizing effect of RAD51 inhibition in glioblastoma stem-like cells
Source: BMC Cancer. 2016 Aug 5;16:604. doi: 10.1186/s12885-016-2647-9 (PMC4974671; doi:10.1186/s12885-016-2647-9)
Supplement: Additional file 4: Figure S1. — Measurement of DNA damage and RAD51 expression in H9-NSC cells. A) H9-NSC line was irradiated at 4Gy and subjected to comet assay at indicated time. Data are given as a percentage of olive tail moment (OTM) and normalized to control (***p < 0.001 versus control cells). B) mRNA expression of RAD51 in group 1, group 2 and H9-NSC (*p < 0.05). The vertical scatter plot shows the log10 expression of relative quantification (RQ) values normalized to the expression before IR. Each data point represents each cell line measured in triplicate. C) H9-NSC viability was measured using an MTS assay after 5 days of RI-1 treatment. D) Comet assay was performed on H9-NSC treated for 24 h with 10 μM of RI-1 before 4Gy IR. Data are given as a percentage of olive tail moment (OTM) normalized to control. (PPTX 326 kb) [file 12885_2016_2647_MOESM4_ESM.pptx]

## Slide 1
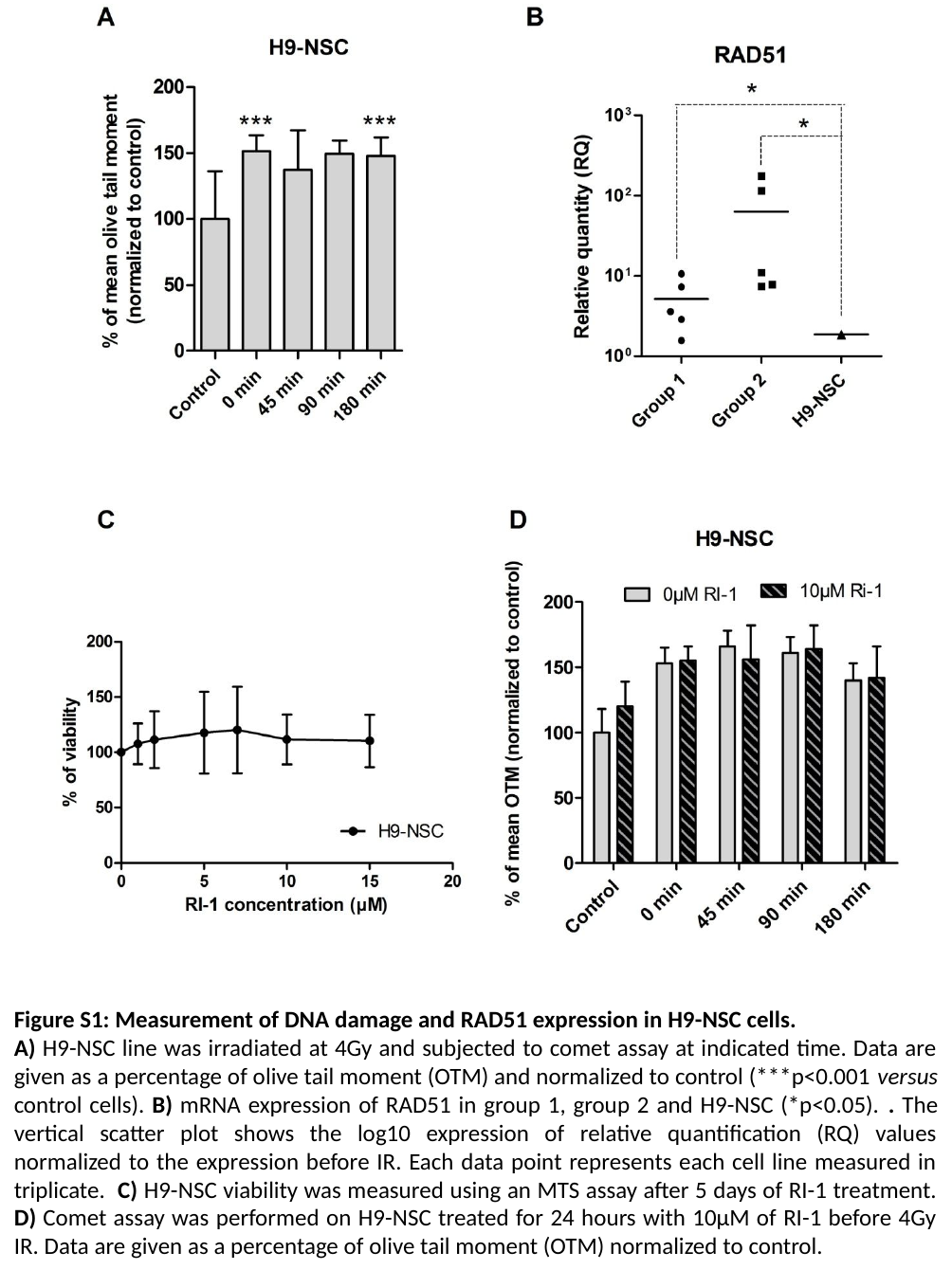

Figure S1: Measurement of DNA damage and RAD51 expression in H9-NSC cells.
A) H9-NSC line was irradiated at 4Gy and subjected to comet assay at indicated time. Data are given as a percentage of olive tail moment (OTM) and normalized to control (***p<0.001 versus control cells). B) mRNA expression of RAD51 in group 1, group 2 and H9-NSC (*p<0.05). . The vertical scatter plot shows the log10 expression of relative quantification (RQ) values normalized to the expression before IR. Each data point represents each cell line measured in triplicate. C) H9-NSC viability was measured using an MTS assay after 5 days of RI-1 treatment. D) Comet assay was performed on H9-NSC treated for 24 hours with 10µM of RI-1 before 4Gy IR. Data are given as a percentage of olive tail moment (OTM) normalized to control.
