# Supplementary material for: A radiosensitizing effect of RAD51 inhibition in glioblastoma stem-like cells
Source: BMC Cancer. 2016 Aug 5;16:604. doi: 10.1186/s12885-016-2647-9 (PMC4974671; doi:10.1186/s12885-016-2647-9)
Supplement: Additional file 5: Figure S2. — Representative comet images of A) GSC-11 (group 1) and B) GSC-14 (group 2) after RI-1 treatment and 16Gy IR (t = 180 min) Comet images were captured with the Axio Imager M2 fluorescent microscope (Carl Zeiss) at 20× (scale bar 100 μm). IR, Ionizing radiation (PPTX 280 kb) [file 12885_2016_2647_MOESM5_ESM.pptx]

## Slide 1
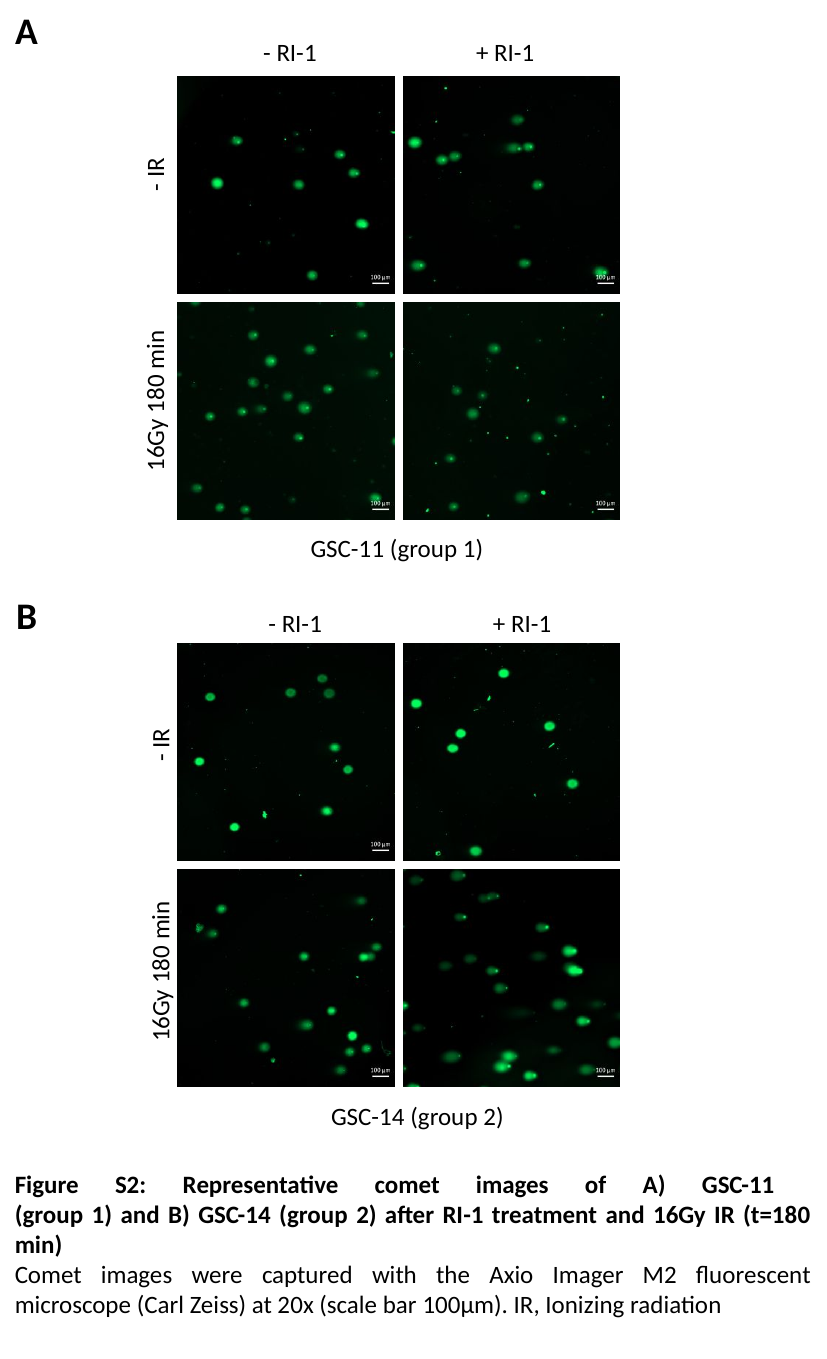

A
- RI-1
+ RI-1
- IR
16Gy 180 min
GSC-11 (group 1)
B
- RI-1
+ RI-1
- IR
16Gy 180 min
GSC-14 (group 2)
Figure S2: Representative comet images of A) GSC-11
(group 1) and B) GSC-14 (group 2) after RI-1 treatment and 16Gy IR (t=180 min)
Comet images were captured with the Axio Imager M2 fluorescent microscope (Carl Zeiss) at 20x (scale bar 100µm). IR, Ionizing radiation
