# Supplementary material for: A radiosensitizing effect of RAD51 inhibition in glioblastoma stem-like cells
Source: BMC Cancer. 2016 Aug 5;16:604. doi: 10.1186/s12885-016-2647-9 (PMC4974671; doi:10.1186/s12885-016-2647-9)
Supplement: Additional file 6: Figure S3. — Relation between patients outcome and radiosensitivity of GSCs. GSCs from group 1 and group 2 are described in the results section; PFS, Progression-free survival. (PPTX 94 kb) [file 12885_2016_2647_MOESM6_ESM.pptx]

## Slide 1
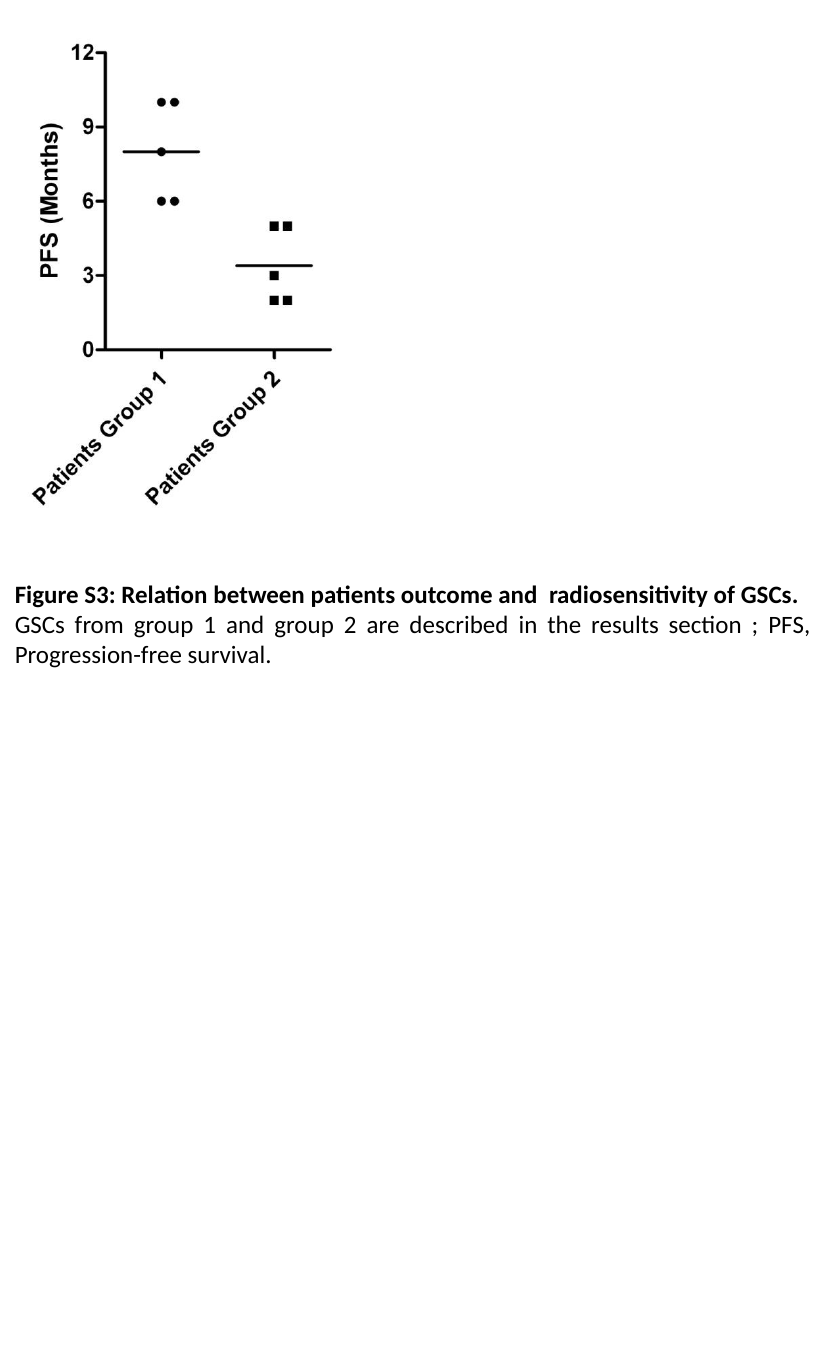

Figure S3: Relation between patients outcome and radiosensitivity of GSCs.
GSCs from group 1 and group 2 are described in the results section ; PFS, Progression-free survival.
